# Avian Influenza Surveillance in the Danube Delta Using Sentinel Geese and Ducks

**DOI:** 10.1155/2014/965749

**Published:** 2014-03-25

**Authors:** Alexandru Coman, Daniel Narcis Maftei, Razvan M. Chereches, Elena Zavrotchi, Paul Bria, Claudiu Dragnea, Pamela P. McKenzie, Marissa A. Valentine, Gregory C. Gray

**Affiliations:** ^1^Center for Health Policy and Public Health, Institute for Social Research, Faculty of Political, Administrative and Communication Sciences, Babes-Bolyai University, 400132 Cluj-Napoca, Romania; ^2^Department of Infectious Diseases, St. Jude Children's Research Hospital, Center of Excellence for Influenza Research and Surveillance, Memphis, TN 38105, USA; ^3^College of Public Health and Health Professions, and Emerging Pathogens Institute, University of Florida, 101 S. Newell Dr., Suite 2150A, Gainesville, FL 32610, USA

## Abstract

Highly pathogenic avian influenza (HPAI) H5N1 virus incursions from migrating birds have occurred multiple times in Romania since 2005. Beginning in September 2008 through April 2013, seasonal sentinel surveillance for avian influenza A viruses (AIVs) using domestic geese (*Anser cygnoides*) and ducks (*Anas platyrhynchos*) in the Danube Delta was established by placing 15 geese and 5 ducks at seven sites. Tracheal and cloacal swabs, and sera collections (starting in 2009) were taken monthly. We studied a total of 580 domestic birds and collected 5,520 cloacal and tracheal swabs from each and 2,760 sera samples. All swabs were studied with real-time reverse transcription polymerase chain reaction (rRT-PCR) for evidence of AIV. Serological samples were studied with hemagglutination inhibition assays against avian H5, H7, and H9 influenza viruses. From 2009 to 2013, 47 swab specimens from Cot Candura, Enisala, and Saon screened positive for AIV; further subtyping demonstrated that 14 ducks and 20 geese had cloacal evidence of H5N3 carriage. Correspondingly, 4 to 12 weeks after these molecular detections, sentinel bird sera revealed elevated HI titers against H5 virus antigens. We posit that domestic bird surveillance is an effective method to conduct AIV surveillance among migrating birds in delta areas.

## 1. Introduction

Asian-lineage highly pathogenic avian influenza (HPAI) H5N1 virus has infected poultry in many countries of the world and is thought to be highly endemic in certain delta areas due to frequent viral incursions from large populations of migrating birds [1, 2]. As of 2011, the Food and Animal Organization (FAO) considered HPAI H5N1 to be enzootic among aquatic birds in Bangladesh, China, Egypt, India, Indonesia, and Vietnam [3]. The latest report from the World Organization for Animal Health revealed outbreaks of HPAI H5N1 that have occurred in Bangladesh, Bhutan, Cambodia, China, India, Korea, Nepal, and Vietnam in 2013 and in China, Nepal, and Vietnam during early 2014 [4].

Large populations of aquatic migratory birds frequent delta areas in Romania and evidence to date strongly suggests that they have introduced HPAI strains into Romania's poultry. The first Romanian HPAI H5N1 incursions were detected in backyard poultry farms in October 2005 [5, 6]. The virus rapidly spread throughout the country until aggressive control measures were taken; the epizootic halted in July 2006 [7]. Laboratory studies demonstrated that the epidemic virus was similar to H5N1 viruses previously detected in Southeast Asia and Turkey and that migrating birds were the likely source [8]. A second HPAI H5N1 outbreak took place in November 2007 and was contained in Murighiol, Tulcea County, Romania [9]. A third HPAI H5N1 detection occurred among poultry in March 2010 in six backyard poultry farms in Letea and in one backyard poultry farm in Plauru, Tulcea County [10]. These two villages are also located in the Danube Delta. As wild bird die-offs occurred in the Danube Delta before, or in concert with these outbreaks, the authors of this paper reasoned that surveillance among migrating birds in the Danube Delta might serve as an early warning system for future avian influenza virus (AIV) incursions.

As HPAI viruses are thought to originate from low pathogenic avian influenza (LPAI) viruses, it is more strategic to conduct surveillance for LPAI viruses, particularly among wild birds before viruses enter domestic flocks. As opposed to HPAI viruses, where signs of disease occurrence are often more visible due to high mortality rates, LPAI often fails in causing overt signs of disease; thus, surveillance can be challenging particularly among wild birds.

Traditional wild bird LPAI surveillance systems often involve substantial costs for labor, trapping equipment, transportation, and laboratory studies. Also, a number of factors can bias results; for example, certain birds are easier to trap than others and seasonality can influence the composition of the wild bird population; hence, trapping during certain time periods can influence the overall sample diversity [11]. Furthermore, trapping of wild birds can inflict injury.

In contrast, monitoring domestic geese and ducks that mix with wild birds in a sentinel system has numerous advantages. Domestic birds are quite sensitive in picking up LPAI from wild birds. Surveillance costs are less as these geese and ducks are easier to sample and sampling can be recurrent so as to identify new LPAI introductions over time. Detections from such domestic geese and duck sentinel systems help to identify viruses that are prone to move into domestic birds before they become enzootic. Such “early warning” detections of LPAI in the aquatic environment can help public health officials initiate interventions to prevent the spread of LPAI to poultry farms.

With these advantages in mind, we established sentinel domestic geese and duck surveillance at geographically diverse sites within the Danube Delta. This report documents early results from this sentinel network.

## 2. Materials and Methods

### 2.1. Location

**Figure 1:**
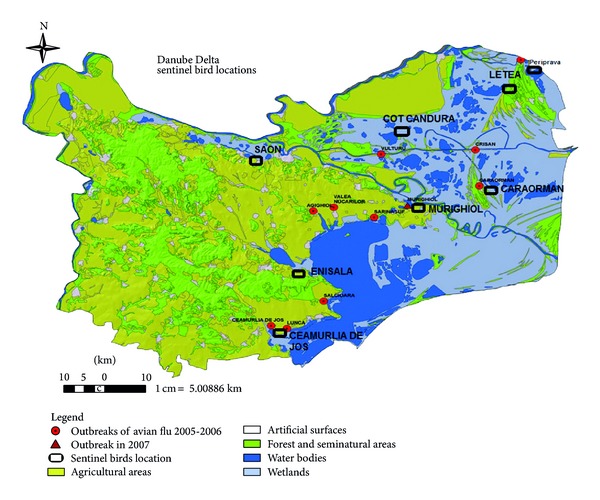
Sentinel bird surveillance sites in the Danube Delta are represented by black rectangles, Tulcea County, Southeastern Romania. In 2011, the Periprava site was discontinued and the Letea site was added.

The Danube Delta is the second largest river delta in Europe, after the Volga Delta. The total delta area is approximately 4,125 km^2^ of which approximately 85% is situated in Tulcea County, Romania. In 2008, we initiated active sentinel domestic geese (*Anser cygnoides*) and duck (*Anas platyrhynchos*) surveillance in the Danube Delta; surveillance was first initiated in one location, Cot Candura, and then expanded to seven sites by August 2009 (Figure 1).

**Figure 2:**
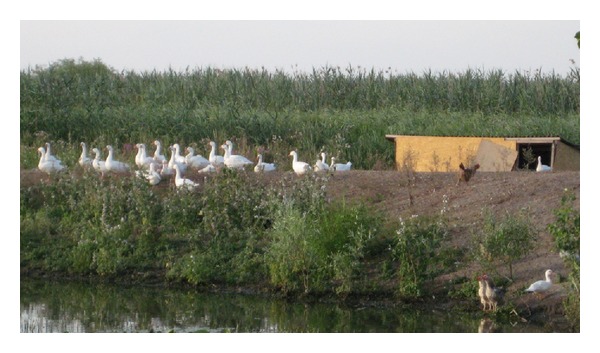
Photograph of a typical sentinel bird shelter, Caraorman site, Danube Delta, September 2010.

**Figure 3:**
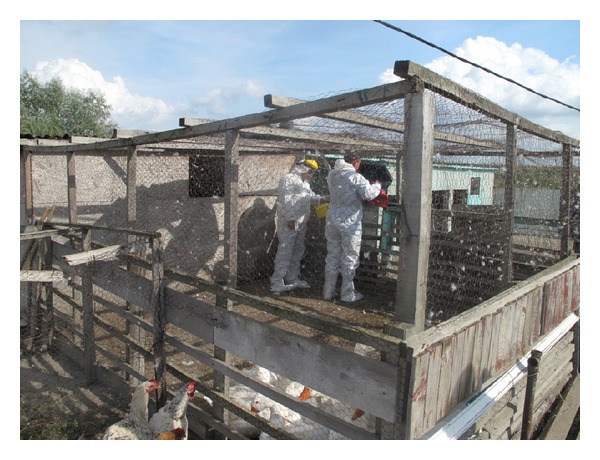
Photograph of a sentinel bird surveillance site, Danube Delta, October 2013.

In an effort to use representative sites for sentinel bird surveillance, researchers selected sites that were geographically diverse, while also taking into account the geographic density of wild bird populations in different areas of the Danube Delta. Specific locations near wetlands or lakes were targeted where previous AIV had been isolated from dead waterfowl or where primary reservoir species frequently gather [12]. In the surveillance sites, shelters and open fenced enclosures (Figures 2 and 3) were constructed to hold 20 domestic birds. The surveillance sites were located at (1) Saon (N45° 13′ 0′′, E28° 33′ 0′′), (2) Ceamurlia de Jos (N44044′9′′, E28043′26′′), (3) Periprava (N45021′05′′, E29037′44′′) replaced in 2011 by Letea (N45010′05′′, E29000′49′′), (4) Cot Candura (N45014′ 52′′, E29004′44′′), (5) Caraorman (N45004′28′′, E29024′55′′), (6) Murighiol (N45000′51′′, E29007′37′′), and (7) Enisala (N44052′55′′, E28048′59′′) (Figure 1).

### 2.2. Bird Selection and Sampling

Surveillance was initiated in 2008 and is still in progress at the time of this report (February 2014). Each year in early September, 140 young, free ranging domestic waterfowl were purchased and 20 were placed at each surveillance site (~15 geese and 5 ducks). Sentinel geese and duck placement during this time period coincides with wild bird migration into the Danube Delta. Both geese and ducks were used to increase the probability of contact with migrating wild geese and duck species. Geese were chosen since they tend to lead the flock back to the pens in the evening, after a day spent on the lakes. The numbers of males to females (1 : 4) were chosen in order to avoid fighting among males. Beginning in the third year of the surveillance, sentinel birds were banded such that the identity and health of each bird could be tracked over the influenza season. Prior to bird banding, in order to ensure the naivety of the flock, any flock that had a positive swab sample was removed and replaced with verified, healthy birds. This was accomplished via testing birds for influenza A virus using real-time RT-PCR (rRT-PCR) and serology to ensure that they were naïve to influenza A viruses prior to release at sentinel sites.

The sentinel birds were free to move around in local waters and to mix with wild waterfowl. Each evening the sentinel birds were encouraged with food to return to the protective enclosure. Nearly every month, cloacal and tracheal swabs were collected by a veterinary team, which traveled to the sentinel sites via boat or car.

The veterinary team also collected blood samples from a random selection of half of the sentinel birds in order to determine their serologic status. Birds that were lost, seropositive, or rRT-PCR positive for influenza were replaced. The birds were tended by local villagers/fishermen who agreed to assist in the surveillance study.

### 2.3. Ethical Statement

Actions were taken to ensure animal health and well-being throughout the study via enlisting licensed public health veterinarians from the Tulcea County Health Department to collect sera and swabs from the sentinel birds. Additionally, the study was approved by the Institutional Animal Care and Use Committee (IACUC) at the University of Florida.

### 2.4. Laboratory Work

Tests were conducted in accordance with the Diagnostic Manual for Avian Influenza of the European Union [13] and the OIE Manual of Diagnostic Tests and Vaccines for Terrestrial Animals (World Organization for Animal Health, 2011). RNA was extracted from swabs using viral RNA kits (Qiagen, Alameda, CA, USA) and analyzed by rRT-PCR targeting the influenza A virus matrix gene. All positive samples were sent to the National Reference Laboratory for Avian Influenza, Bucharest, Romania, followed by the Weybridge EU/OIE/FAO reference laboratory for further investigation (virus isolation, sequencing, and genotyping). Positive samples were also sent to the Global Pathogens Laboratory at the University of Florida for molecular study. The detection of new AIV strains was quickly communicated to the local and central veterinary authority.

### 2.5. Serological Assays

From September 2009 through March 2013, serological samples were obtained from a random selection of half of the sentinel birds at each location. From September 2009 through September 2011, serological samples were characterized using an ELISA blocking kit for AIV type A antibody detection (Pourquier Institute, now part of Idexx Laboratories, Inc., Westbrook, ME, USA). Positive sera were then tested using a hemagglutination inhibition (HI) assay. Beginning in September 2011, only the HI test was performed. All serological testing was conducted in the Tulcea County Sanitary Veterinary and Food Safety Laboratory. H5N2, H7N3, and H9N2 avian influenza antigens (Istituto Zooprofilattico Sperimentale delle Venezie, Legnaro, Italy) were used in the HI assays. The study used these antigens for screening since H5, H7, and H9 influenza strains have caused AIV outbreaks in humans and in birds.

## 3. Results

Cloacal and tracheal swabs collected from each sentinel bird (5,520 each) and sera samples collected from a subset of sentinel birds (2,760) between September 2008 and April 2013 and September 2009 and April 2013, respectively, were screened for evidence of AIV infection. From 2009 through 2013, 47 swabs from 47 birds at 3 distinct locations screened positive for influenza A virus on five different occasions (Table 1). After subtyping the virus, results revealed 34 cloacal swabs positive for H5N3. H5N3 virus was present in 14 ducks and 20 geese, although no clinical signs were observed in sentinel or wild birds from the sentinel sites. The remaining 13 influenza A rRT-PCR positive samples from Enisala and Saon were confirmed by the Romanian National Reference Laboratory and the Global Pathogens Laboratory at the University of Florida, respectively; however, no virus was isolated or sequenced from the remaining 13 samples due to low viral load. H5N3, detected in 2009 at Cot Candura, was the first LPAI virus ever isolated in Romania. Each new AIV strain detected was quickly communicated to both the local and national veterinary authorities.

In the subset of previously immunologically naïve sentinel birds, serological assays revealed elevated HI titers against H5, H7, and H9 viruses (Table 2). We found elevated HI antibodies against the H5N2 antigen within 4–12 weeks after the first molecular detections of H5N3 virus. Approximately 8 weeks after the HI elevations, antibody titers waned to levels <1 : 16.

## 4. Discussion

National AIV surveillance programs within the European Union vary in terms of the resources and the types of surveillance methods used [14]. Four main surveillance strategies are employed: (1) active surveillance involving testing of live caught, wild birds, (2) active surveillance of hunted birds, (3) active surveillance (periodic sampling) using sentinel, domestic birds kept in high-risk areas, and (4) passive surveillance and laboratory study of dead, wild birds or poultry when unusual mortality is detected [15]. Active surveillance involving wild caught birds proves suitable for detecting both HPAI and LPAI viruses in high-risk areas, while active surveillance of hunted birds has frequently been successful in detecting LPAI viruses. Use of the hunted bird approach however does not permit sampling for the entire respiratory season. Active surveillance of sentinel domestic birds is used infrequently but has been useful in both HPAI and LPAI virus detections. Romania relies upon the passive surveillance strategy among domestic and wild birds and hence, AIV surveillance is sparse and dependent upon the discovery and study of sick or dead wild birds. For comparison purposes, the Romanian passive system of surveillance did not uncover any evidence of LPAI viruses during the study period. When analyzing financial costs, use of methods 1 and 2 add additional financial constraints due to the requirement of additional traps and net cannons, prolonged time spent in the field, as well as the need for additional boats and gas. These incurred costs would supplement those already incurred by the sentinel surveillance method.

Among the sentinel geese and ducks included in the current surveillance study, we detected several LPAI H5N3 viruses and found serological evidence that LPAI H7 and H9 virus strains were also circulating (Table 2). The detection of elevated and rapidly reduced serologic titers against LPAI virus suggests that these infections may be subclinical, transient, and missed by periodic tracheal and cloacal swabs followed by molecular assays alone.

Detection of LPAI in sentinel bird populations serves as an important precursor for preventing HPAI outbreaks. Prior to HPAI outbreaks, circulating AIVs undergo mutations and can transform into LPAI, oftentimes without causing overt mortality in flocks until it mutates into a HPAI. The public health response to both LPAI and HPAI remains the same: this includes culling infected birds. In LPAI situations, this prevents the spread of LPAI and contact with existing viruses in animals, which could result in mutation and hence, potential emergence of HPAI. Thus, surveillance for LPAI in bird populations remains a key component in helping to prevent HPAI outbreaks.

During the study period, only 2 documented H5N1 outbreaks occurred within birds in the Danube Delta—one in Letea and one in Plauru. The outbreaks occurred in 2010, within 2 weeks of each other. We posit that the sentinel surveillance did not detect H5N1 incursions since the distance between the closest sentinel site and the outbreak area was 15 km. In 2011, in order to enhance study detection, the Periprava site was discontinued and a new sentinel site in Letea was established.

## 5. Conclusion

We propose that domestic geese and ducks can be used as an effective AIV sentinel surveillance system, especially in delta areas where the domestic birds can freely mix with large populations of migrating birds. Using this method, we were able to detect the presence of H5N3 via molecular surveillance as well as find serological evidence of circulating H7 and H9 viruses. Additionally, for our sentinel bird surveillance, we estimated that costs were less expensive than using other AIV surveillance methods such as trapping, hunting, or collecting wild bird carcasses. Use of sentinel bird surveillance may also provide a more representative example of circulating viruses due to the species biases that accompanies hunting or sampling from deceased birds. Thus, domestic bird surveillance is less invasive, less expensive, and more effective than using other surveillance strategies.

## Figures and Tables

**Table 1:** Number and location of real-time RT-PCR influenza A detections among sentinel birds in Romania's Danube Delta, 2009–2013.

Date	Collection site	Type of sample	Number of positive samples/total number of tested samples/year (%)
01/2009	Cot Candura	Cloacal swabs	10/400 (2.5%)
09/2010	Cot Candura	Cloacal swabs/tracheal swabs	20/400 (5%)
10/2011	Enisala	Cloacal swabs	4/400 (1%)
03/2013	Saon	Cloacal swabs/tracheal swabs	9/400 (2.25%)
03/2013	Cot Candura	Cloacal swabs/tracheal swabs	4/400 (1%)

**Table 2:** Geese or duck sera with elevated serum hemagglutination inhibition assays against H5, H7, and H9 hemagglutinin antigens. Collection period: 2010–2013.

Collection site	Hemagglutinin inhibition assay (% elevated)*		
	H5 (%)	H7 (%)	H9 (%)
Ceamurlia de Jos	1 : 32 (1%)		
Cot Candura	1 : 256 (7.7%)	1 : 128 (1.3%)	
Enisala	1 : 16 (3%)		
Letea	1 : 16 (2.5%)	1 : 64 (1%)	
Saon	1 : 16 (4%)	1 : 16 (1%)	
Caraorman			1 : 128 (0.5%)

*A titer ≥1 : 16 was considered positive; total number of sera samples collected = 300/collection site.
